# Remembering Sir Nicholas White

**DOI:** 10.1186/s12936-026-05842-y

**Published:** 2026-03-12

**Authors:** Frank Smithuis, Elizabeth Ashley, Mallika Imwong, Arjen Dondorp, Francois Nosten, Kesinee Chotivanich, Nick Day, Sasithon Pukrittayakamee

**Affiliations:** Mahidol-Oxford Tropical Medicine Research Unit (MORU), Faculty of Tropical Medicine, Mahidol University, Bangkok, PO 10400, Thailand


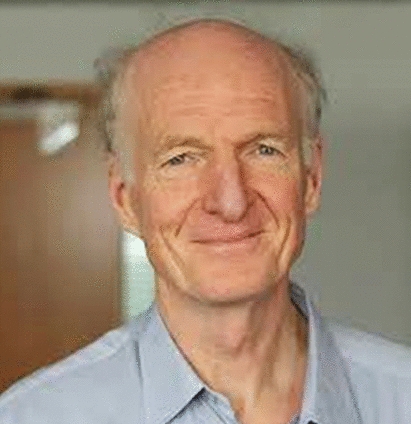
We are deeply saddened by the death of Professor Nick White. Nick was one of the most remarkable, versatile and intellectually gifted individuals of our time.

 First and foremost, he was widely respected for his brilliance in medical science. He possessed an extraordinary and uniquely broad understanding of clinical medicine, biology, pathophysiology, pharmacology, epidemiology and mathematics. By integrating these disciplines, he achieved an exceptionally depth of insight into infectious diseases, and had the rare ability to generate innovative ideas, study them and ensure they led to practical solutions that could be delivered to those who needed them most. He was internationally recognised as the leading expert in uncomplicated and severe malaria, most notably for his pivotal role in introducing antimalarial drugs derived from qinghaosu (artemisinin), a traditional Chinese herb, that Chinese scientists had identified as active against resistant malaria parasites.

Beyond malaria, his expertise extended to many other diseases including melioidosis, enteric fever, rickettsiosis, tetanus, dengue haemorrhagic fever, Japanese encephalitis, tuberculosis and COVID.

He greatly expanded Mahidol Oxford Research Unit (MORU), a research institute in Thailand, and established multiple new research units in Vietnam (the Oxford University Clinical Research Unit, OUCRU), as well as in other countries across the region and beyond. These centres became highly productive and internationally renowned, supporting a broad network of researchers, working in partnership with hospitals, and clinicians around the world.

Nick’s research focused on practical solutions that had maximum impact on health outcomes in resource-limited settings. He was an activist at heart. The driving force behind his scientific work and advocacy was a humanitarian desire to improve the lives of those in need. This conviction nurtured his tireless fighting spirit and determination, particularly when confronting those who resisted the call for better science and more effective treatments for people in low income countries, whose voices are often unheard. It was this unwavering resolve that translated scientific evidence into tangible results, which ultimately defined Nick’s legacy. To name a few;

In the 1990s Nick developed the theory for artemisinin-based combination treatment and led groundbreaking studies that provided evidence of their superior effectiveness. These treatments were rapidly acting, safe and well tolerated. Over the following decade, he fought a long and determined battle to persuade conservative forces within WHO, USAID, and other institutes, who were in favor of continued use of failing first-line malaria treatments such as chloroquine and sulfadoxine-pyrimethamine. Across large parts of the world, these drugs had lost their efficacy, and their continued use was contributing to a devastating rise in malaria-related mortality, costing countless lives. Through persistence, scientific rigor, and moral conviction, not accepting substandard treatment, Nick helped drive the global transition to artemisinin-based therapies, as the first-line treatment for *Plasmodium falciparum* malaria, and to injectable artesunate for the treatment of severe malaria. These treatments have since been used in more than a billion patients and saved millions of lives.

More recently Nick strongly criticized WHO’s decision to halt the use of rectal artesunate suppositories as a pre-referral treatment of children with severe malaria in remote or inaccessible areas where most deaths occur. This decision was based on preliminary results of one observational study. Nick and colleagues analysed the study and argued that the design and analysis were questionable, and did not provide sufficient evidence to halt such an important and potentially life-saving drug. The WHO has since retracted its decision.

Nick was unafraid to challenge research findings that he considered implausible. A recent example involved two COVID-19 related articles on chloroquine, published in *The Lancet* and *The New England Journal of Medicine*. Nick and his colleagues quickly identified inconsistencies in the data, investigated the studies and demonstrated that the published data, and thus the conclusions, were incorrect. They then brought this scandal to light, and both articles were retracted following their investigation.

He fought his battles with intellectual rigor and superior expertise, which earned him deep respect among many colleagues, but it also created adversaries. After many years of service, he was removed from his advisory role with the WHO Global Malaria Programme, and he was invited back only following a change in the Programme’s leadership.

Nick also raised the issue of corruption in global health, recognizing its destructive impact, felt most severely by the world’s poor, and suggesting that it could be the single greatest obstacle to improvement. “The subject of corruption is usually avoided, being too sensitive, too difficult, too depressing and perhaps too serious. Somehow, we need to be able to talk about corruption. And then we need to measure it and devise counter-measures. It is not intractable. In recent years discussions on racial and gender discrimination in the international health sector have been constructive and transformative. The pervasive problem of substandard and falsified medicines and tests has been brought to light and is starting to be addressed”.

Even his funders were not exempt from criticism. Nick had been supported by the Wellcome Trust for more than four decades and was sincerely grateful for its longstanding support, both in building the research networks in Thailand and Vietnam and his fellowship funding, that enabled him to pursue his own research interests. Recently, however, he became concerned about a shift in the Trust's philosophy toward a more top-down approach, and he expressed the hope that the perspectives and experience of researchers working in low-resource settings would continue to play a central role in shaping its direction. 

Nick was a passionate, principled and tenacious fighter throughout his career. His uncompromising commitment to improving science and health care for resource-poor populations consumed much of his time and energy, continuing until the very last days of his life.

Nick was an extremely hard worker and productive scientist. He trained and inspired hundreds of scientists and students, strengthening local research capacity, particularly in Asia, but also in Africa and South America. He had a rare gift for making even the most complex theories accessible, explaining them with clarity and simplicity, that empowered others to grow. Many of those he mentored went on to become leaders in their own right. He authored or co-authored more than 1,300 scientific publications and over 50 book chapters, making him one of the most prolific and highest cited researchers in infectious diseases worldwide. When Nick’s name appeared as a co-author, it truly meant something. He made substantial contributions to nearly every article. His support was intense and unwavering. He frequently conceived the original idea and went in detail through the study design. After the study was completed, he often refined the graphs, suggested important additions and thoroughly edited the manuscript. Through his level of engagement, he not only elevated the quality of the work, but also mentored his students, who learned a great deal and significantly improved their research skills under his guidance.

Beyond the importance of rigorous science, Nick firmly believed that research findings had meaning only when they were translated into real-world impact for the populations most in need. He was a strong supporter of medical aid organizations and advocacy institutions, serving on the boards of several and generously supporting some of them as a donor.
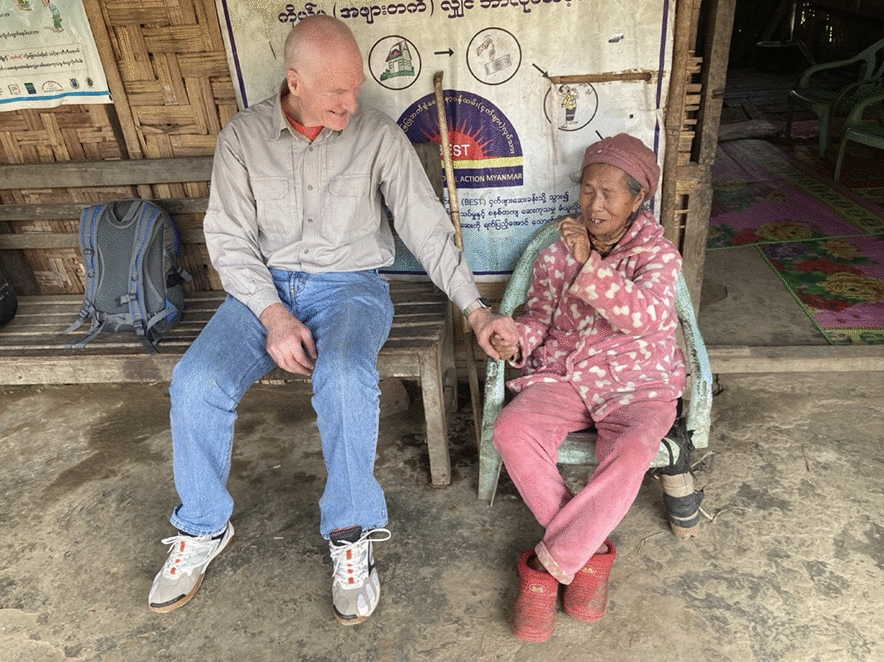


*Picture taken during a visit to the Community Health Worker program of Medical Action Myanmar in Putao,* a *remote area in the eastern Himalayan region, in the far north of Myanmar, 2024.*

Beyond his professional life, Nick had a wide range of interests and passions. He was a gifted and fiercely competitive sportsman. He played squash at a high level and was widely regarded as one of the finest cricketers in Thailand. He served as vice-captain when Thailand played its first overseas international cricket match in 1991, defeating a strong Hong Kong side in Kowloon. He was also a devoted member of the British Club’s “fun” side, where losing was almost more fun than winning. True to his mischievous wit, after the team finally won, ending a two-year stretch of 34 consecutive defeats, Nick admonished them *"This is a disgrace—we were on the verge of a world-record breaking string of successive defeats matched only by the Pitcairn Islands second XI. Lads should be ashamed of themselves. Now we have to start again…".* His last games were played in Chang Mai and Kuala Lumpur in 2022 at the age of 70, still commanding respect from the opposition.

Nick was known for his playful and infectious sense of humor. He was also a talented cartoonist, creating illustrations for the cricket team, MORU Christmas cards and at many other moments that called for a smile.

He was musical, playing both guitar and harmonica. He loved the blues and would often join jam sessions at Brown Sugar and The Saxophone, two of Bangkok’s best-known blues bars.

Nick loved nature, and expressed this by purchasing plots of land where he planted trees, creating small nature reserves. He knew countless species and took pleasure in understanding their characteristics as well as those of the animals that roamed around in them.

His kindness was the hallmark of his life. He was always seeking ways to help others, whether in their scientific careers, their work, or their health and well-being. If you were sick, he worried. He would visit you if he could, or call regularly to ask about your symptoms, and suggest treatments. He sincerely worried about our safety, while we worked in conflict-ridden areas and regularly offered advice on how to reduce risks.

Working with Nick was a blessing. His scientific brilliance, his enthusiasm and inspiration, combined with his honesty, kindness and his infectious sense of humor, made him an extraordinary mentor and an endearing friend. We will miss him deeply.

Our heartfelt condolences go to his wife, daughters, family and friends.


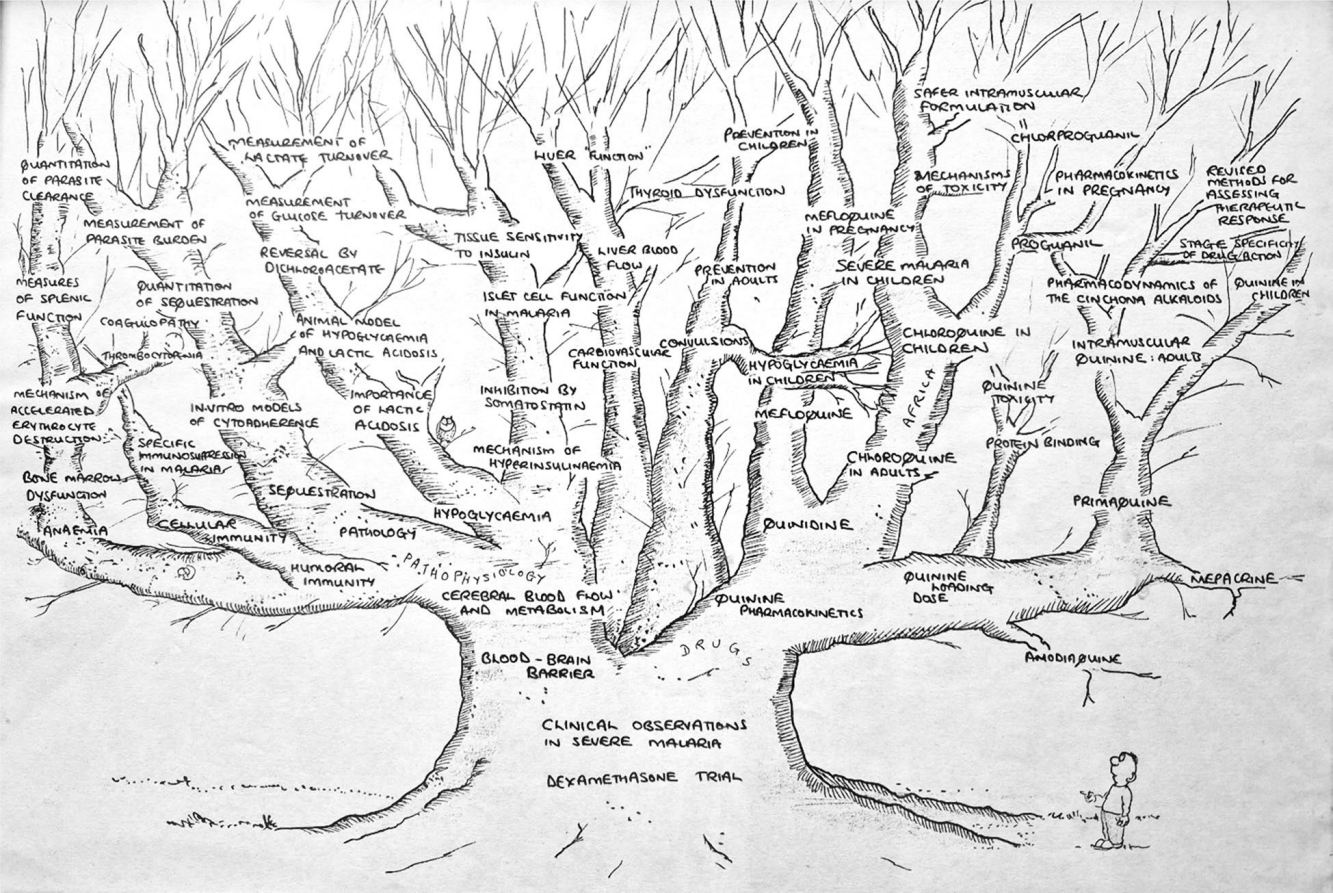


A cartoon Nick drew in the 90'ies, summarizing his thinking about malaria those days.

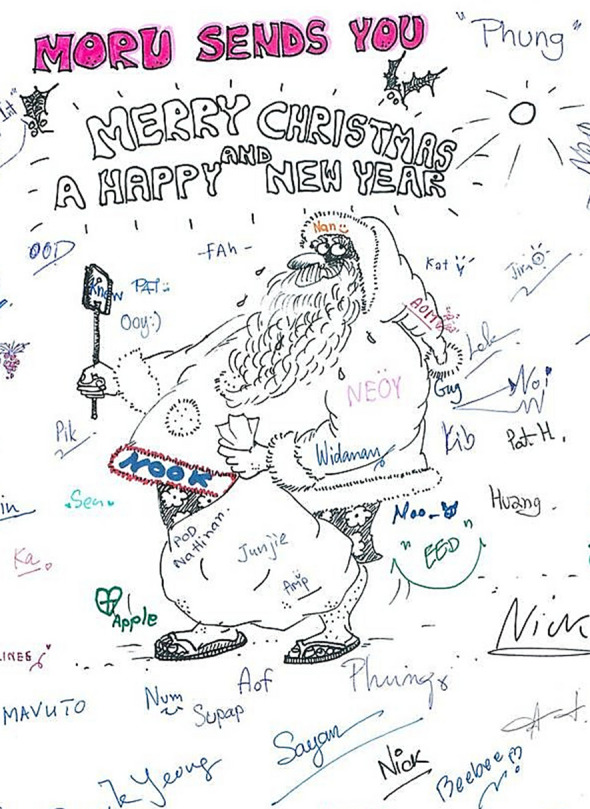



*One of Nick's Christmas/New Year cartoons*

