# Correction: Remembering Sir Nicholas White

**DOI:** 10.1186/s12936-026-05905-0

**Published:** 2026-04-29

**Authors:** Frank Smithuis, Elizabeth Ashley, Mallika Imwong, Arjen Dondorp, Francois Nosten, Kesinee Chotivanich, Nick Day, Sasithon Pukrittayakamee

**Affiliations:** Mahidol-Oxford Tropical Medicine Research Unit (MORU), Faculty of Tropical Medicine, Mahidol University, Bangkok, 10400 Thailand

**Correction:** **Malaria Journal (2026) 25: 136**

10.1186/s12936-026-05842-y

Following publication of the original article [1], the following authors and their affiliation were omitted from the author list, now have been added to the article.

Elizabeth Ashley^1^, Mallika Imwong^1^, Arjen Dondorp^1^, Francois Nosten^1^, Kesinee Chotivanich^1^, Nick Day^1^ and Sasithon Pukrittayakamee^1^

Original article [1] has been updated.
